# Impact of post-harvest storage conditions on polyphenol composition and antioxidant activity in natural almonds

**DOI:** 10.3389/fnut.2025.1582434

**Published:** 2025-04-23

**Authors:** Agnese Ragno, Martina Imbesi, Teresa Gervasi, Antonella Smeriglio, Giuseppina Mandalari, Daniela Impellizzeri, Domenico Trombetta

**Affiliations:** ^1^Department of Chemical, Biological, Pharmaceutical and Environmental Sciences (ChiBioFarAm), University of Messina, Messina, Italy; ^2^Department of Biomedical and Dental Sciences and Morphofunctional Imaging, University of Messina, Messina, Italy

**Keywords:** natural almonds, microbial control, natural almond skin, storage, polyphenols, antioxidant activity

## Abstract

**Background:**

Post-harvest storage of natural almonds is a critical step, as it can cause the onset of microbial contamination and modify polyphenolic composition of almond skin, potentially altering the antioxidant capacity and overall, the health effects of the native product. This study aims to evaluate the impact of different post-harvest storage conditions on the microbiological stability, polyphenolic profile, and antioxidant activity of natural almonds (*Prunus dulcis* cv. “Fascionello”).

**Methods:**

Natural almonds were obtained from *Consorzio Mandorla di Avola* and stored under three conditions: light exposure at room temperature (RT), dark at RT, and dark at 4°C, monitoring temperature and relative humidity. Samples were analyzed at four time points (T_0_, T_3_, T_6_, and T_9_ months). Microbiological stability was assessed using standard plate-counting techniques; polyphenolic content was determined through spectrophotometric assays and RP-LC-DAD-ESI-MS/MS analysis, whereas the antioxidant activity was evaluated using different spectrophotometric and spectrofluorimetric assays including DPPH, TEAC, FRAP, and ORAC assays. Chemometric analyses were performed to compare polyphenolic changes across different storage conditions over time.

**Results:**

Temperature remained stable with moderate variations, indicating a well-controlled environment, while humidity exhibits significant fluctuations, likely influenced by external factors. No significant microbial contamination was detected throughout storage, confirming the microbiological safety of natural almonds. The polyphenolic content significantly decreased within the first 3 months, particularly under dark conditions at RT. However, a recovery phase was observed at 6 months, with cold-stored almonds retaining the highest levels of total polyphenols and flavonoids. The antioxidant activity of almond skin extracts correlated with polyphenolic content, showing an initial decline followed by stabilization under refrigerated storage. Hierarchical clustering analyses highlighted distinct polyphenolic expression patterns based on storage conditions and time points.

**Conclusion:**

Post-harvest storage conditions significantly impact the polyphenolic profile and antioxidant properties of natural almonds. For short-term storage (≤6 months), RT with controlled light exposure is sufficient to preserve polyphenols, whereas cold and dark conditions are recommended to maintain bioactive compound stability and antioxidant potential for long-term storage (>6 months). These findings provide valuable insights for optimizing storage strategies in the food, nutraceutical, and pharmaceutical industries.

## 1 Introduction

Almonds (*Prunus dulcis* Mill. DA Webb) are among the most versatile and nutritious foods in the world, consumed both raw and in multiple industrial transformations, such as gluten-free flours and dairy alternatives. Their cultivation, primarily concentrated in regions with a Mediterranean climate, such as California, Spain, Australia, and southern Italy, has experienced exponential growth in recent decades, thanks to the global demand for healthy and functional foods (1).

The high consumption of almonds, coupled with their proven nutraceutical properties, has attracted the attention of many researchers, not only in the agronomic sector, but also in the biomedical and pharmaceutical fields (2).

From a nutritional perspective, almonds are a health powerhouse: rich in α-tocopherols, fiber, proteins, and fats, mainly unsaturated, they contribute to cardiovascular health and weight management (3, 4). In addition to their health benefits, the valorization of by-products derived from almond processing (such as shells, peels, and bleaching water) presents a crucial challenge to enhance the sustainability of the sector, reduce waste, and promote the circular economy (5).

Several studies have demonstrated that almond peel is an excellent source of dietary fiber, lipids, and proteins, and contains a high concentration of phenolic compounds. These studies indicate that the total polyphenol content in almond peel extracts is about 10 times higher than that in whole seed extracts (6). Although the polyphenolic profile may vary largely based on the origin of the almonds and the specific variety investigated (7, 8), almond skins contain mainly flavan-3-ols, flavonols, flavanones as well as hydroxybenzoic and hydroxycinnamic acids (6). These compounds exhibit various activities, including antioxidant, anti-inflammatory, antiviral, and anticancer effects (9).

The antioxidant activity of phenolic compounds is mainly due to their structural characteristics (benzene ring and the number/position of hydroxyl groups), based on oxidation–reduction metabolic reactions. These compounds can eliminate oxidizing species, absorb metal ions by forming metal complexes with their multiple hydroxyl and carbonyl groups, interfere with the activity of inducible nitric oxide synthase (iNOS), mitigating oxidative stress caused by NO, inhibit the activity of xanthine oxidase (XO) and NADPH Oxidases (NOX) enzymes, and enhance the activation of antioxidant enzymes such as superoxide dismutase (SOD), catalase (CAT), and glutathione peroxidase (GPx) (10).

Moreover, polyphenols have been shown to have synergistic effects with vitamins C and E, protecting against oxidation and improving antioxidant defense (6).

However, polyphenols are unstable molecules and, therefore, can degrade and/or react with elements such as oxygen and metal ions during processing and storage, leading to changes in their structure and biological activities (11).

Literature highlights that physical and environmental factor, such as temperature, humidity, and exposure to light as well as almond genetic features and industrial processing, can significantly contribute to the degradation of phenolic compounds (12).

Post-harvest storage of natural almonds is the first critical step, as it can cause the onset of microbial contamination and modify polyphenolic composition of almond skin, potentially altering the antioxidant capacity and in general the health effects of the product (12–14). Despite studies regarding lipid degradation, sensory changes and microbial contaminations of natural almonds are available (13, 15, 16), studies about the polyphenol’s behavior and antioxidant activity change over time during simulated-post-harvest storage of natural almonds are still lacking.

Some studies have demonstrated that nuts polyphenols content can increase over time, depending on industrial processing and storage conditions. In one study, for example, processed almonds stored for 15 months at 4 and 23°C showed an increase in the levels of flavonoids and phenolic acids in almond skin. The same study also showed that accelerated aging with elevated temperature and humidity resulted in a higher number of polyphenols (12). Another study, which has shown that low temperatures preserve the polyphenol content of walnuts and their antioxidant activity, supported these results (17).

Given these considerations and the lacking literature on microbial contamination, polyphenolic content, and antioxidant activity of raw Sicilian almonds under controlled storage conditions, we aimed to investigate these aspects in detail. Specifically, this study was designed to assess: (i) the microbial contamination of natural almonds supplied by the *Consortium Mandorla di Avola*; (ii)the total polyphenolic content of almond natural skin (NS) extracts; (iii)the changes in their phytochemical profile over different time points (T_0_, T_3_, T_6_, and T_9_ months) and under varying storage conditions (including light exposure, dark, and dark at 4°C and also tracking the relative humidity levels) by reverse-phase liquid chromatography coupled with diode array detection and electrospray ionization tandem mass spectrometry (RP-LC-DAD-ESI-MS/MS). Indeed, RP-LC-DAD-ESI-MS/MS provides an advanced and sensitive approach for the identification and quantification of polyphenolic compounds in almond skin, enabling comprehensive profiling of bioactive molecules. Moreover, the application of multivariate statistical analysis allows for the interpretation of complex datasets, revealing correlations and patterns within the polyphenolic profile, critical for understanding the nutritional and health-related properties of almond skin such as the antioxidant properties, here investigated by cell-free based models.

## 2 Materials and methods

### 2.1 Sample collection and storage conditions

Natural almonds (*Prunus dulcis*, cv. “Fascionello”) were kindly provided by *Consorzio Mandorla di Avola* and harvested in 2023. The experiment began with the opening of vacuum-sealed samples and the collection_._ of an initial 0.5 kg portion to carry out the first extraction at time 0 (T_0_). Concurrently, whole almonds were distributed into three distinct storage conditions for subsequent extractions at 3-, 6-, and 9-months post-harvest (T_3_, T_6_, and T_9_). Each storage condition included 1.5 kg of almonds, which were stored as follows: (i) Light exposure at room temperature (Light RT, L): almonds were placed in a wide, shallow tray to minimize overlap and positioned on a bench near a window, ensuring exposure to natural daylight; (ii) Dark at room temperature (Dark RT, D): almonds were stored in a closed, opaque box in the same room as the Light RT samples, ensuring the same ambient temperature but without light exposure; (iii) Cold storage in dark (Dark 4°C, D4): almonds were stored in a sealed opaque container inside a refrigerator maintained at 4°C. For both room temperature storage conditions (Light RT and Dark RT), temperature and relative humidity were monitored weekly using a digital thermometer/hygrometer (ThermoPro) to track environmental fluctuations over the storage period. At each time point (T_3_, T_6_, T_9_), almonds from each condition were thoroughly mixed before collecting a 0.5 kg sample for further processing. The collected almonds were then subjected to the extraction procedures described in the following section 2.3.

### 2.2 Effect of different storage conditions on the microbiological stability of almonds

Almond samples stored under different conditions were tested for their microbiological stability according to the following official regulations and guidelines (18–21).

Plate counting of total mesophilic bacteria, fungi (yeasts and molds), total coliforms, *Escherichia coli*, Enterobacteria, *Staphylococcus aureus*, and Sulphite-Reducing Clostridia (SRB) was performed.

All media were supplied by ThermoFisher Scientific (Oxoid Ltd., Basingstoke, UK). Samples were homogenized with PBS (pH = 7.4) mixing vigorously, serial dilutions were then performed and aseptically spread over the media plates. Specifically, plate count agar (PCA) was used for mesophilic counts and incubated at 30 ± 2 h °C for 72 h ± 2 h; malt extract agar with 10% lactic acid (MEA) was used for yeasts and molds and incubated at 30°C for 72 h; Enterobacteriacee were investigated using Violet Red Bile Glucose Agar (VRBGA) using an incubation period of 48 ± 2 h at 37 ± 2 h °C.

Coliforms and *E. coli* were analyzed by the standard membrane filter technique using Chromogenic Coliform Agar (CCA) and Tryptone Bile X-Gluc agar (TBX) with incubation period of 24 h at 37°C and of 18/24 h at 44°C.

The standard membrane filter technique using Sulfite Polymyxin Sulfadizine agar (SPS) was used for the determination of SRB after an incubation at 37°C for 48 h.

All microbial analyses were carried out in triplicate.

### 2.3 Sample preparation and extraction

Each batch of natural almonds was cryo-peeled using liquid nitrogen through repeated freeze–thaw cycles, manually removed, and then crushed in the presence of liquid nitrogen using an analytical mill (Model A 11 BASIC IKA) according to Mandalari et al. (22). The obtained natural skin (NS) powder underwent three steps of defatting process using n-hexane (70 ml) under continuous stirring for 6 h. The resulting residue was treated with 100 ml of a methanol–HCl 0.1% (v/v) solution and extracted three times by sonication for 15 min each. The collected supernatants were pooled and dried using a rotary evaporator (Hei-VAP Core, Heidolph Instruments GmbH & Co., Schwabach, Germany). The remaining pellet was dissolved in 40 ml of deionized water and extracted with 40 ml of ethyl acetate; a process repeated four times. The collected organic phases were left on an anhydrous sodium sulphate bed for 20 min and subsequently dried using the above rotary evaporator. The average extraction yield obtained was 1.64%. The light, dark and dark 4°C (L, D and D4, respectively) dried NS extracts at different time points (T_0_, T_3_, T_6_ and T_9_) were stored in a vacuum desiccator in the dark. For analysis, the extracts were freshly solubilized in methanol for phytochemical and *in vitro* cell-free tests, or in DMSO for cell-based assays.

### 2.4 Phytochemical analyses

#### 2.4.1 Total phenolic compounds

The total phenolic compounds were determined following the method described by Ingegneri et al. (8). Briefly, 10 μl of the test extracts (0.3125–2.5 mg/ml) or methanol as blank, were added to 90 μl of deionized water and 100 μl of Folin–Ciocalteu reagent, followed by a 3-min incubation. Subsequently, 50 μl of 10% sodium carbonate was added, and the samples were incubated in the dark at room temperature for 1 h, vortexing every 10 min. Samples were then plated in 96-well microplate, and absorbance was measured at 785 nm using a Multiskan™ GO Microplate Spectrophotometer (Thermo Fisher Scientific, Waltham, MA, USA). Gallic acid (0.075–0.6 mg/ml) was used as the reference standard, and results were expressed as grams (g) gallic acid equivalents (GAE) per 100 g of dry extract (DE).

#### 2.4.2 Flavonoids

Total flavonoid content was quantified according to the method described by Lenucci et al. (23) by using rutin as the reference standard (0.625–0.5 mg/ml). Briefly, 50 μl of the test extracts (0.3125–2.5 mg/ml) were added to 450 μl of deionized water. Subsequently, 30 μl of a 5% NaNO₂ solution was added, and the samples were incubated at room temperature (RT) for 5 min. After incubation, 60 μl of a 10% AlCl₃ solution was added, followed by a 6-min incubation. Finally, 200 μl of 1 M NaOH and 210 μl of deionized water were added, and the samples were vortex mixed. Absorbance was recorded at 510 nm (UV–VIS Spectrophotometer UV-1601, Shimadzu Italia S.r.l.) using methanol as blank. The results were expressed as g of rutin equivalents (RE) per 100 g of DE.

#### 2.4.3 RP-LC-DAD-ESI-MS/MS analysis

The variations in the polyphenolic composition of extracts obtained from different batches of almonds stored under various conditions were analyzed using RP-LC-DAD-ESI-MS/MS, following the method described by Danna et al. (24) with some modifications. Chromatographic separation was performed on a Luna Omega PS C18 column (150 mm × 2.1 mm, 5 μm; Phenomenex, Torrance, CA, USA) at 25°C, using a mobile phase consisting of 0.1% formic acid in water (Solvent A) and acetonitrile (Solvent B). The elution gradient was programmed as follows: from 0 to 3 min, Solvent B was set at 0%; from 3 to 9 min, it increased to 3%; from 9 to 24 min, it reached 12%; from 24 to 30 min, it was raised to 20% and maintained at that level until 33 min. Between 33 and 43 min, Solvent B was increased to 30%, followed by a further increase to 50% from 43 to 63 min, where it remained until 66 min. Between 66 and 76 min, Solvent B was set at 60% and held constant until 81 min. Finally, from 81 to 86 min, Solvent B was returned to 0%, followed by a 4-min re-equilibration phase. A 5 μl injection volume was used for analysis. UV–Vis spectra were recorded over a wavelength range of 190–600 nm, with chromatograms acquired at 260, 292, 330 and 370 nm to ensure comprehensive identification of polyphenol classes. Mass spectrometric analysis was conducted using an Agilent 6,320 ion trap mass spectrometer (Agilent Technologies, Santa Clara, CA, USA) operating in negative ionization mode (ESI−). The instrument parameters were set as follows: capillary voltage at 3.5 kV, nebulizer pressure (N₂) at 40 psi, drying gas temperature at 350°C, drying gas flow rate at 9 L/min, and skimmer voltage at 40 V. Mass spectra were acquired using a fragmentation energy of 1.2 V (MS/MS). Data acquisition was performed in full-scan mode (90–1,000 m/z), and the collected data were processed using Agilent ChemStation software (version B.01.03) and Agilent Trap Control software (version 6.2). Analytes identification was carried out by comparing the retention times, UV–Vis, MS and MS/MS spectra of each analyte with those of commercially available HPLC-grade reference standards (see Table 1), as well as with literature data and free online consulting UV–Vis and mass spectra databases (SpectraBase®, PhytoHub, ReSpect for Phytochemicals, Mass Bank and PubChem). The abundance of polyphenols in total ion current chromatogram was expressed as ion peak intensity. To minimize variability due to instrument drift or ionization fluctuations, all almond NS extracts were analyzed within a single LC–MS sequence, without any inter-day variation and under strictly controlled and consistent conditions, to ensure reliability and internal consistency of the data.

**Table 1:** Tentative identification of polyphenolic profile of natural almond skin (NS) extracts by RP-LC-DAD-ESI-MS/MS analysis.

Compounds	[M − H]^−^ (m/z)	MS/MS	λ_max_ (nm)
Hydroxybenzoic acids			
Protocatechuic acid^*^	153	109; 81	258; 293
Hydroxycinnamic acids			
Chlorogenic acid^*^	353	191; 179; 173; 135	245; 325
trans-p-Coumaric acid^*^	163	119; 93	309
Ferulic acid	193	178; 149; 134	320
Flavanones			
Naringenin-7-O-glucoside^*^	433	271; 151	284
Eriodictyol-7-O-glucoside^*^	449	287; 151; 119	284
Naringenin^*^	271	151; 119	288
Eriodyctiol^*^	287	151; 125	288
Flavonols			
6,8-Dihydroxykaempferol	317	299; 273; 151; 135	264; 367
Quercetin-3-O-galactoside^*^	463	301; 179; 151	257; 370
Quercetin-3-O-rutinoside^*^	609	463; 301; 179; 151	257; 370
Quercetin-3-O-rhamnoside	447	301; 179; 151	257; 370
Quercetin-3-O-glucoside^*^	463	301; 179; 151	257; 370
Isorhamnetin-3-O-rutinoside^*^	623	477; 315; 300; 151	257; 368
Isorhamnetin-3-O-glucoside^*^	477	315; 300; 165; 151	254; 368
Kaempferol-3-O-galactoside	447	285; 151; 117	264; 367
Kaempferol-3-O-rutinoside^*^	593	447; 285; 151; 117	264; 367
Kaempferol-3-O-glucoside	447	285; 151; 117	264; 367
Kaempferol	285	257; 229; 151; 117	264; 367
Quercetin^*^	301	273; 257; 179; 151	257; 370
Isorhamnetin	315	300; 179; 151; 133	257; 368
Flavanols			
Catechin^*^	289	245; 205;179	279
Epicatechin^*^	289	245; 203; 179; 151	279
(+)-GC 3-O-gallate^a^	457	305; 169; 151	275
4’-O-MEGC O-glucuronide ^b^	495	319;304; 179; 151	284; 323
3’-O-Methyl-epicatechin	303	288; 259; 205; 179; 151	275
EGC O-glucuronide ^c^	481	305; 261; 179; 151	284; 323

a (+)-Gallocatechin 3-O-gallate.

b 4’-O-methyl(epi)gallocatechin O-glucuronide.

c (Epi)gallocatechin O-glucuronide.

* Checked with commercially available HPLC-grade reference standards (purity ≥ 98%) purchased from Extrasynthase (Genay, France).

### 2.5 Antioxidant activity

#### 2.5.1 DPPH

The 2,2-diphenyl-1-picrylhydrazyl (DPPH) scavenging activity was assessed following the method described by Ingegneri et al. (8). In a 96-well plate, 150 μl of a freshly prepared 1 mM DPPH solution in methanol was combined with 3.75 μl of test extracts (15–120 μg/ml) or methanol as blank. The mixture was incubated at RT for 20 min, after which absorbance was recorded at 517 nm using the plate reader specified in Section 2.3.1. Trolox (2.5–20 μg/ml) was used as the reference standard.

#### 2.5.2 TEAC

The radical scavenging activity against 2,2′-Azino-bis (3-ethylbenzothiazoline-6-sulfonic acid) (ABTS) was determined following the method described by Ingegneri et al. (8). A water-based ABTS^•⁺^ radical solution was prepared by mixing 1.7 mM ABTS•⁺ with 4.3 mM ammonium persulfate in a 1:5 (v/v) ratio. The mixture was incubated in the dark at RT for at least 12 h to allow radical formation. Before use, the solution was diluted with deionized water to achieve an absorbance of 0.7 ± 0.02 at 734 nm and used within 4 h. For the assay, 10 μl of the test sample solution (7.5–60 μg/ml) or methanol as blank, were added to 200 μl of the ABTS^•⁺^ radical solution. The mixture was incubated for 6 min at RT, after which absorbance was measured at 734 nm using the same plate reader described in Section 2.3.1. Trolox (1.25–10 μg/ml) was used as the reference standard.

#### 2.5.3 FRAP

The Ferric Reducing Antioxidant Power (FRAP) assay was conducted following the method described by Ingegneri et al. (8). The reagent was prepared by mixing 300 mM acetate buffer (pH 3.6), 10 mM 2,4,6-tris(2-pyridyl)-s-triazine (TPTZ) dissolved in 40 mM HCl, and 20 mM FeCl₃. For the assay, 200 μl of the reagent was combined with 10 μl of the test samples (2.5–240 μg/ml) or methanol as blank, directly in a 96-well plate. The reaction mixture was incubated in the dark for 4 min, after which absorbance was measured at 593 nm using the same plate reader described in Section 2.3.1. Trolox (1.25–10 μg/ml) was used as the reference standard.

#### 2.5.4 ORAC

The Oxygen Radical Absorbance Capacity (ORAC) assay was performed following the method described by Ingegneri et al. (8). The experiment was conducted in a 96-well fluorescence plate, where 120 μl of 117 nM fluorescein was combined with 20 μl of the test samples (0.03125–2.5 μg/ml) or methanol as blank, which were diluted in 75 mM phosphate-buffered saline (PBS) at pH 7.4. The plate was incubated in the dark at 37°C for 15 min. After incubation, 60 μl of 40 mM 2,2′-azobis(2-methylpropionamidine) dihydrochloride (AAPH) was added to initiate the reaction. The fluorescence signal was recorded every 30 s for 90 min using a plate reader (FLUOstar Omega, BMG LABTECH, Ortenberg, Germany), with an excitation wavelength of 485 nm and an emission wavelength of 520 nm. Trolox (0.25–1.875 μg/ml) was used as the reference standard.

### 2.6 Statistical analysis

The data were presented as IC_50_ values with their respective 95% confidence limits or as the mean ± standard deviation (S.D.) from three independent experiments in triplicate (*n* = 3). Statistical significance was assessed using a one-way analysis of variance (ANOVA), followed by the Student–Newman–Keuls post-hoc test. A *p*-value ≤ 0.05 was considered statistically significant. Data analysis was performed using SigmaPlot 12.0 software (Systat Software Inc., San Jose, CA, USA).

Chemometric analyses, including dendrogram, hierarchical clustering analysis (HCA) and 2D correlation heatmap, were conducted with JMP7 software (SAS Institute Inc., Cary, NC, USA). To compare differences between the different polyphenolic profiles and storage conditions, the Euclidean distance was calculated, and hierarchical clustering analysis was performed using Ward’s variance-minimization method.

## 3 Results

### 3.1 Trend analysis of temperature and humidity over time

**Figure 1:**
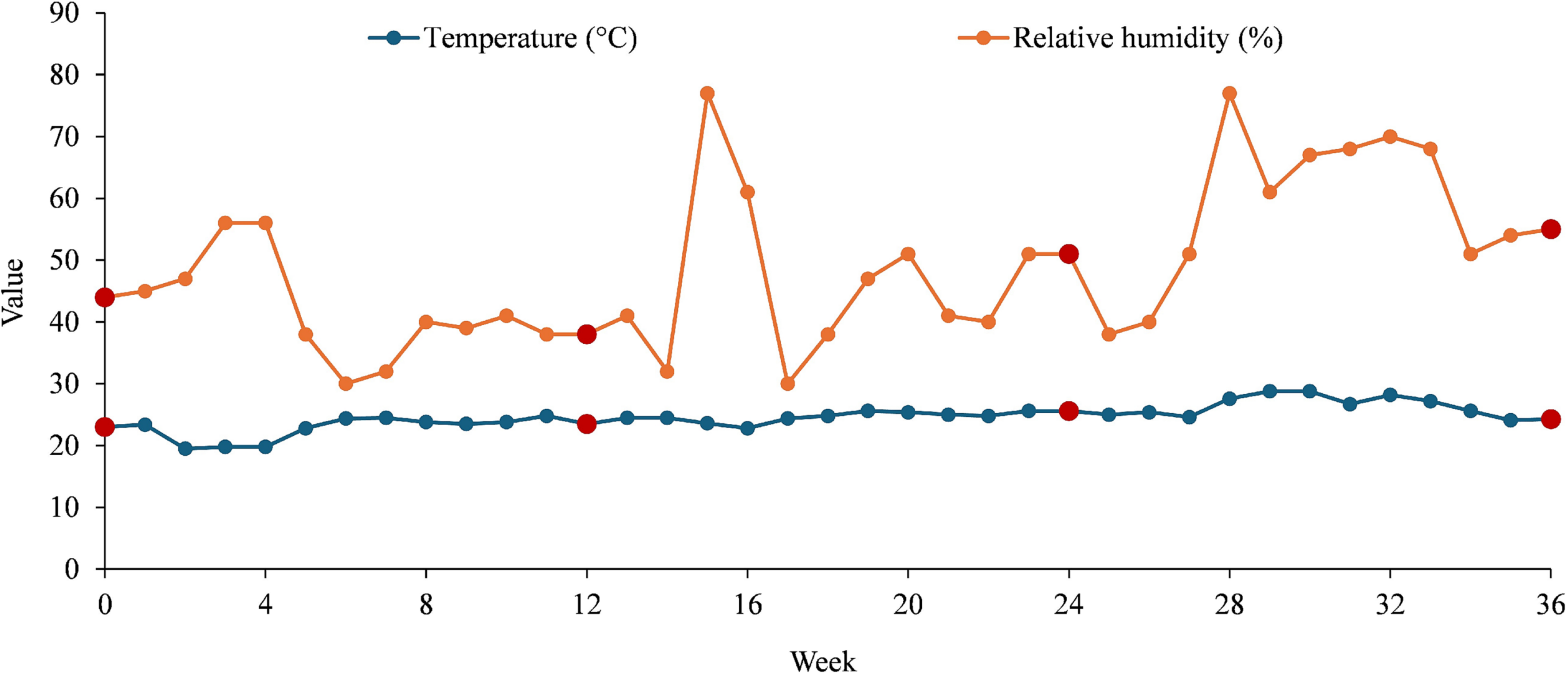
Temperature and humidity trends over time (36 weeks) with collecting points highlighted in dark red.

The analysis of temperature and humidity trends revealed important insights into environmental conditions (Figure 1).

The recorded mean temperature of 24.59°C indicated a moderate climate with relatively stable conditions over time. The median temperature (24.55°C) being very close to the mean suggests that temperature variations follow a symmetrical distribution, with no extreme deviations. The standard deviation of 2.15°C confirms that fluctuations were minimal, meaning that temperature remained consistent throughout the observed period. The range between 19.5 and 28.8°C showed a moderate variation of 9.3°C, which could be attributed to seasonal changes.

Unlike temperature, humidity showed greater variability. The mean humidity of 48.58% falls within the comfortable range for indoor environments. However, the median humidity of 46%, slightly lower than the mean, that certain weeks recorded higher humidity levels, skewing the distribution. The standard deviation of 13.06% highlights significant fluctuations, indicating a less stable humidity environment compared to temperature. The range from 30 to 77% suggests that external factors, such as seasonal humidity changes or weather conditions, play a crucial role in influencing moisture levels.

Temperature remains stable over time, with only moderate variations, suggesting a well-controlled environment with limited external interference. Humidity, on the other hand, fluctuates significantly, which may be caused by external climate conditions. The increase in humidity levels, reaching up to 77% in certain weeks, could pose potential issues for moisture-sensitive environments, as it may lead to condensation, mold growth, or material degradation. The weak correlation between temperature and humidity suggests that humidity is influenced by other environmental factors rather than temperature alone. The stability of temperature indicates effective climate control, while humidity fluctuations require further monitoring to ensure consistency in environmental conditions. Understanding these variations is essential for maintaining an optimal indoor environment, particularly in industries where precise climate control is crucial. The recorded values at weeks 0, 12, 24, and 36 (collecting points) offer a more complete perspective on how temperature and humidity evolved over time.

At week 0, the temperature was 23.0°C, and humidity was 44%, indicating stable initial conditions. By week 12, the temperature increased slightly to 23.5°C, while humidity decreased to 38%, suggesting drier conditions possibly due to seasonal or environmental changes. At week 24, the temperature rose further to 25.6°C, and humidity increased to 51%, marking a shift toward warmer and more humid conditions. By week 35, the temperature slightly dropped to 24.1°C, but humidity continued to rise to 54%, suggesting that humidity fluctuates more independently compared to temperature.

This pattern reinforces the idea that temperature remains relatively stable within a narrow range (24.05 ± 1.13°C), while humidity exhibits more significant variations (46.75 ± 7.18%), likely influenced by external factors such as seasonal changes, ventilation, or environmental moisture levels.

### 3.2 Microbiological stability of almond samples

The impact of the different almond storage conditions on the overall microbial counts is reported in Table 2.

**Table 2:** Total mesophile, fungi, coliforms and SRB counts (log CFU/g) in almond samples throughout 9 months storage under varying storage conditions (L, D and D4).

		Sampling time								
	T_0_	T_3_			T_6_			T_9_		
		L	D4	D	L	D4	D	L	D4	D
TMC^a^	3.06·10^2^	5.71·10^2^	1.60·10^2^	4.18· 10^3^	3.00·10^2^	3.06· 10^2^	2.08·10^2^	7.39·10^2^	2.48·10^2^	8.10· 10^2^
Fungi	3.06·10^2^	1.43·10^2^	1.60·10^2^	1.80·10^2^	6.00·10^2^	2.04·10^2^	1.04·10^2^	6.30·10^2^	3.73·10^2^	1.02·10^2^
Enterobacteria	2.04·10	1.00 ·10	7.20·10	ND	ND	ND	ND	ND	ND	ND
Total coliforms	ND	ND	1.90·10	ND	ND	ND	ND	ND	ND	ND
Fecal coliforms	ND	ND	ND	ND	ND	ND	ND	ND	ND	ND
SRB^b^	ND	ND	ND	ND	ND	ND	ND	ND	ND	ND

a TMC, Total mesophile count; bSRB, Sulphite-Reducing Clostridia; ND, not determined.

No significant differences (*p* > 0.05) in microbial growth were detected during storage time, as well as between storage conditions. For the total mesophiles, the microbial count of T_0_ was 3.06 ·10^2^ log CFU/g, slightly increasing after 9 months in the L and D samples, whereas even after 9 months, the number was relatively stable in the D4 condition. A similar trend was observed for fungi, assuming that, the storage conditions did not affect the microbiological properties of the samples. No coliforms were detected, except for almonds stored for 3 months at 4°C in the dark. SRBs were absent in all samples.

According to the Commission Regulation (EC) No. 2073/2005 (25) and its amendment Regulation (EC) No. 1441/2007 (26), which sets legal microbiological criteria for several food products, these nuts could be defined as safe products.

### 3.3 Polyphenol content and modifications in the native phytochemical profile under simulated storage conditions

The initial step involved determining the total phenolic content (TPC) and flavonoid content (TFC) of NS extracts over time (T_0_, T_3_, T_6_, and T_9_) to evaluate the samples’ behavior under simulated storage conditions (light, dark, and dark at 4°C). The results, expressed as grams of gallic acid equivalents (GAE) per 100 g of dry extract (DE) for TPC and as grams of rutin equivalents (RE) per 100 g of DE for TFC, are presented in Table 3. Both the total phenolics and flavonoids content, which are at their highest at T_0_, undergo a sharp decline after 3 months of storage. Surprisingly, the most significant decrease occurs in the dark, whereas the highest polyphenol content is observed in the sample exposed to light, with a statistically significant difference (*p* < 0.001) compared to both DT_3_ and D4T_3_ (Table 3). After an additional 3 months (T_6_), not only is a significant increase in the polyphenolic content of the analyzed extracts observed, but an inversion in the behavior of the samples also occurs. Specifically, D4T_6_ exhibits a significantly (*p* < 0.001) higher content of both total phenolics and flavonoids compared to LT_6_ and DT_6_ (Table 3). After a further 3 months (T_9_), the situation changes again. Indeed, while the D4T_9_ sample maintains the highest total phenolic and flavonoid content, which also increases compared to T_6_, the DT_9_ sample shows a significantly (*p* < 0.001) higher total phenolic content than LT_9_. However, this trend is not observed in terms of total flavonoid content, which remains significantly (*p* < 0.001) lower than LT_9_ (Table 3).

**Table 3:** Quantification of total phenolic compounds (TPC) and total flavonoid content (TFC) using in vitro colorimetric assays in almond natural skin (NS) extracts under different storage conditions.

NS	TPC (g GAE/100 g)	TFC (g RE/100 g)
T_0_	32.3 ± 1.54^a^	26.67 ± 1.05^a^
LT_3_	6.97 ± 0.27^b^	5.36 ± 0.15^e^
DT_3_	3.7 ± 0.11	3.12 ± 0.08
D4T_3_	6.14 ± 0.24	5.29 ± 0.10
LT_6_	14.77 ± 0.38^c^	14.49 ± 0.42^c^
DT_6_	6.1 ± 0.17	5.88 ± 0.16
D4T_6_	18.23 ± 0.44	17.29 ± 0.55
LT_9_	19.34 ± 0.38^d^	15.83 ± 0.32^d^
DT_9_	23.08 ± 0.29	14.1 ± 0.58
D4T_9_	26.96 ± 1.12	17.63 ± 0.19

Measurements were taken at the initial time point (T_0_) and after 3, 6, and 9 months (T_3_, T_6_, and T_9_, respectively) under simulated storage conditions, including light exposure (L), dark (D), and dark at 4°C (D4). Results are expressed as grams of gallic acid equivalents (GAE) per 100 g of NS for TPC and grams of rutin equivalents (RE) per 100 g of NS for TFC. Data represent the mean ± standard deviation (SD).

a *p < 0.001 vs. L, D and D4 T3-9 NS extracts.*

b *p ≤ 0.05 vs. D and D4 T3.*

c *p < 0.001 vs. D and D4 T6.*

d *p < 0.001 vs. D and D4 T9.*

e *p < 0.001 vs. DT3.*

Beyond the total phenolic and flavonoid content, and considering the specific behavior of the examined samples, it was deemed appropriate to analyze the polyphenolic profile using RP-LC-DAD-ESI-MS/MS analysis.

As shown in Tables 1 and 4, a total of 27 compounds were identified, including 4 phenolic acids and 23 flavonoids, in accordance with the findings from the preliminary phytochemical tests (Table 3).

**Table 4:** RP-LC-DAD-ESI-MS/MS analysis of natural almond skin (NS) extracts at different time points (T_0_-T_9_) and under various storage conditions, including light exposure (L), dark (D), and dark at 4°C (D4).

Compounds	Peak Intensity (x 10^5^)									
	T_0_	LT_3_	DT_3_	D4T_3_	LT_6_	DT_6_	D4T_6_	LT_9_	DT_9_	D4T_9_
Hydroxybenzoic acids										
Protocatechuic acid	0.826	1.168	0.631	0.716	1.072		0.919	1.621	1.069	0.663
Hydroxycinnamic acids										
Chlorogenic acid	5.733	1.347			1.387		0.012	15.439	15.204	
trans-p-Coumaric acid	9.268							8.583	11.154	11.871
Ferulic acid	0.697	0.599								
Flavanones										
Naringenin-7-O-glucoside	5.164	5.437	3.168		3.876	2.378	4.864	4.102	4.736	5.439
Eriodictyol-7-O-glucoside	0.675					0.528				0.603
Naringenin	3.413									2.506
Eriodyctiol	7.261						1.207			
Flavonols										
6,8-Dihydroxykaempferol	11.102	0.023				1.619		6.210	7.529	7.352
Quercetin-3-O-galactoside	7.505	2.819	1.104	1.360		1.298			4.705	4.319
Quercetin-3-O-rutinoside	2.516	7.789				1.225				
Quercetin-3-O-rhamnoside	7.804	2.472	1.022		2.924		2.519	5.176	5.825	5.472
Quercetin-3-O-glucoside	8.200	6.238	2.895	5.011	7.182	3.579	6.467	7.249	7.038	7.891
Isorhamnetin-3-O-rutinoside	23.050	23.496	15.282	22.328	22.208	10.946	20.037	19.466	21.333	19.751
Isorhamnetin-3-O-glucoside	36.560	24.299	5.893	12.238	26.407	0.129	24.622	30.710	32.502	31.447
Kaempferol-3-O-galactoside	4.413		1.159					1.942	1.536	2.754
Kaempferol-3-O-rutinoside	11.196	1.679	3.604	1.856	2.624	3.183	6.154	7.731	7.516	8.551
Kaempferol-3-O-glucoside	3.538			1.086			1.603		2.575	2.482
Kaempferol	3.951		1.163					4.341	5.149	5.726
Quercetin	1.440			3.957						
Isorhamnetin	10.247	24.997	23.815	24.627	19.783	23.235		5.972	5.995	5.758
Flavanols										
Catechin	2.565							1.645		3.093
Epicatechin	38.068	9.652	0.880		11.065	3.650	8.030	19.319	24.255	24.483
(+)-GC 3-O-gallate ^a^	0.754		0.655	0.896			0.821	0.736		
4’-O-MEGC O-glucuronide^b^	0.769	0.692	0.697	0.647		0.570			0.666	0.644
3’-O-Methyl-epicatechin	0.895	1.223	0.955	1.174	1.588	0.935	1.559	0.588	0.549	
EGC O-glucuronide^c^	0.916				0.623			0.639	0.928	0.889
Total polyphenols	208.528	113.929	62.924	75.895	100.740	53.276	78.814	141.418	160.266	151.695

The abundance of polyphenols in TIC chromatogram was expressed as ion peak intensity.

a (+)-Gallocatechin 3-O-gallate.

b 4’-O-methyl(epi)gallocatechin O-glucuronide.

c (Epi)gallocatechin O-glucuronide.

The most representative phenolic acids belong to the hydroxycinnamic class, with trans-*p*-coumaric acid and chlorogenic acid being particularly prominent (Table 4). Among flavonoids, flavonols constitute the most numerically represented class, with numerous derivatives of quercetin, kaempferol, and isorhamnetin. These are followed by flavanols, where epicatechin and its derivatives are the most abundant compounds, and finally by flavanones, with naringenin-7-O-glucoside as the most representative compound. To better understand the differences in the expression of these secondary metabolites among the examined samples, the results were expressed as peak intensity, enabling a relative quantitative analysis based on the abundance of the various polyphenolic peaks identified in the total ion current chromatogram. Additionally, the total relative amount of all identified compounds in the NS extracts, recorded over time and under each storage condition, is reported at the end of Table 4. T_0_ exhibits the highest polyphenol content, and the trend observed at T_3_ is fully consistent with the TPC findings. On the contrary, a higher total polyphenols content in LT_6_ compared to D4T_6_, and a higher total polyphenol content in DT_9_ compared to D4T_9_, were detected.

**Figure 2:**
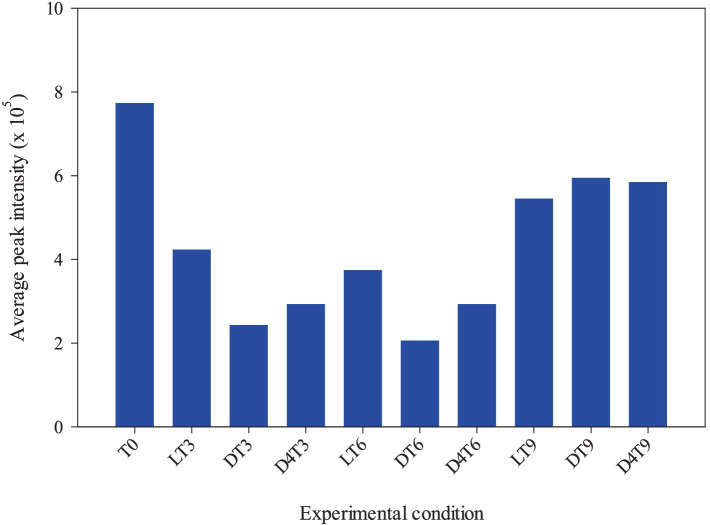
Correlation between the average peak intensity and the different experimental conditions (light RT, dark RT and dark 4°C) over time (T_0_-T_9_).

These discrepancies can likely be attributed to the varying sensitivity of certain compounds, given that the analysis provides a relative rather than an absolute quantification. Indeed, the behavior highlighted by comparing the total polyphenols amount of each time and storage conditions reflects perfectly that showed by comparing the average peak intensity with the different experimental conditions (Figure 2).

**Figure 3:**
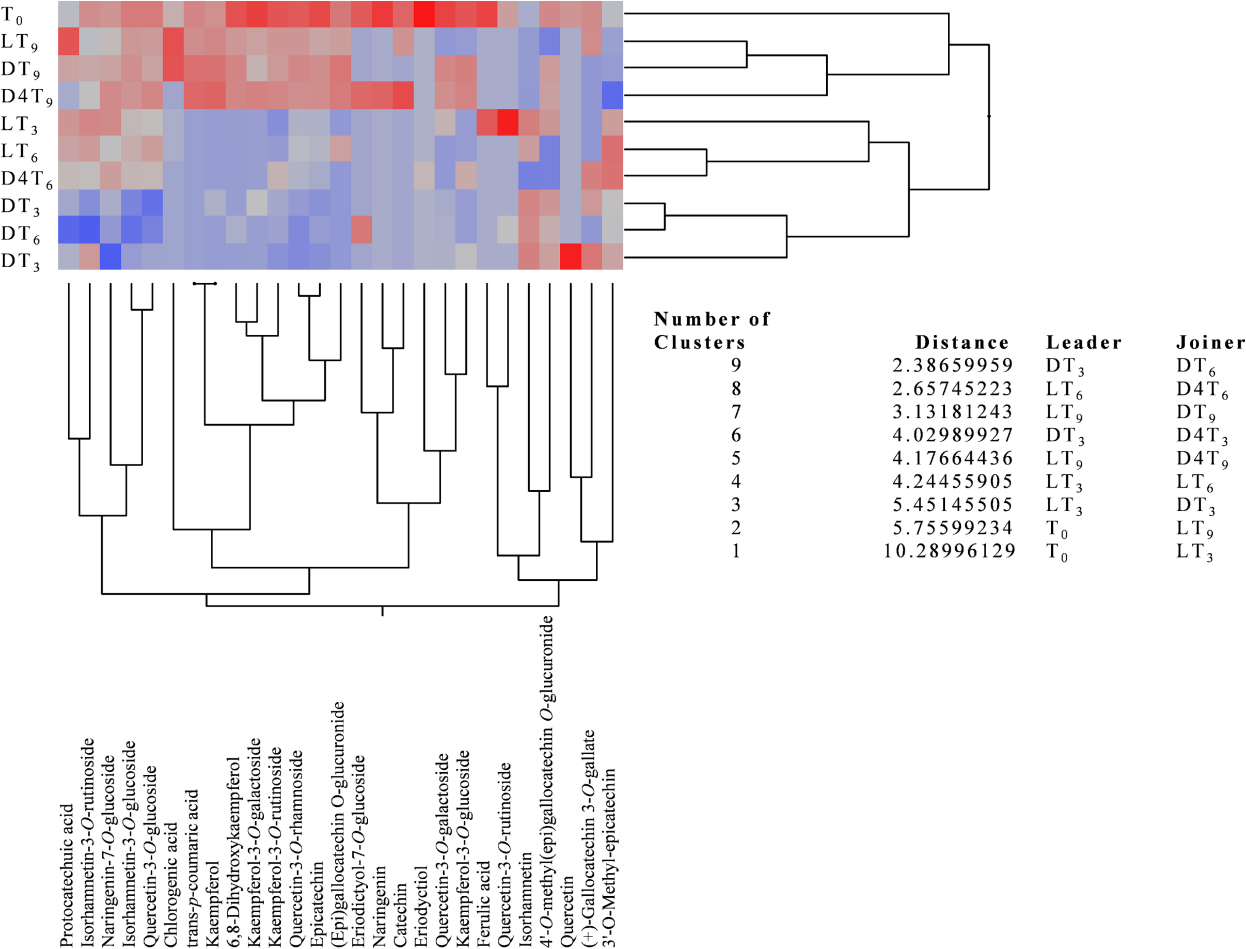
Agglomerative two-way hierarchical clustering analyses of the polyphenolic profiles of natural almond skin (NS) extracts at different time points (T_0_, T_3_, T_6_, and T_9_) and under various storage conditions, including light exposure (L), dark (D), and dark at 4°C (D4).

Beyond the total polyphenol content, it is particularly interesting to observe how the polyphenolic profile evolves over time and in response to different storage conditions. To facilitate the interpretation of these data and highlight statistically significant differences between the various samples, an agglomerative two-way hierarchical clustering analysis was performed (Figure 3).

This analysis is an unsupervised statistical method used to group both samples and variables (in this case, metabolites) based on their similarity, measured using Euclidean distance, to identify meaningful relationships and trends. This bottom-up approach begins with each data point as an individual cluster and progressively merges the most similar clusters until a single hierarchical structure is formed. The analysis produces a heatmap with a dendrogram, facilitating pattern recognition and interpretation. In this visualization, the most highly expressed metabolites are color-coded from dark red to light red, while the less expressed ones range from grey to light blue, with those completely absent represented in dark blue.

The agglomerative two-way hierarchical clustering analysis identified nine clusters, all with distances greater than one, thereby highlighting a statistically significant difference between leaders and joiners across all identified clusters (Figure 3). Specifically, the greater the distance, the more pronounced the differences in the polyphenolic profile, and consequently, the lower the similarity between the examined NS extracts. As shown in Figure 3, DT_3_ and DT_6_ exhibit the greatest similarity in terms of polyphenolic profile; however, they are also the most dissimilar when compared to T_0_. Conversely, D4T_3_ demonstrates a higher expression of secondary metabolites, making it comparable to DT_3_ but with a greater distance. In order of similarity to T0 and polyphenolic expression, samples D4T_9_, DT_9_, and LT_9_ exhibit the highest resemblance, along with the greatest expression of secondary metabolites. An intermediate behavior in terms of secondary metabolite expression was observed for LT_3_ and LT_6_.

**Figure 4:**
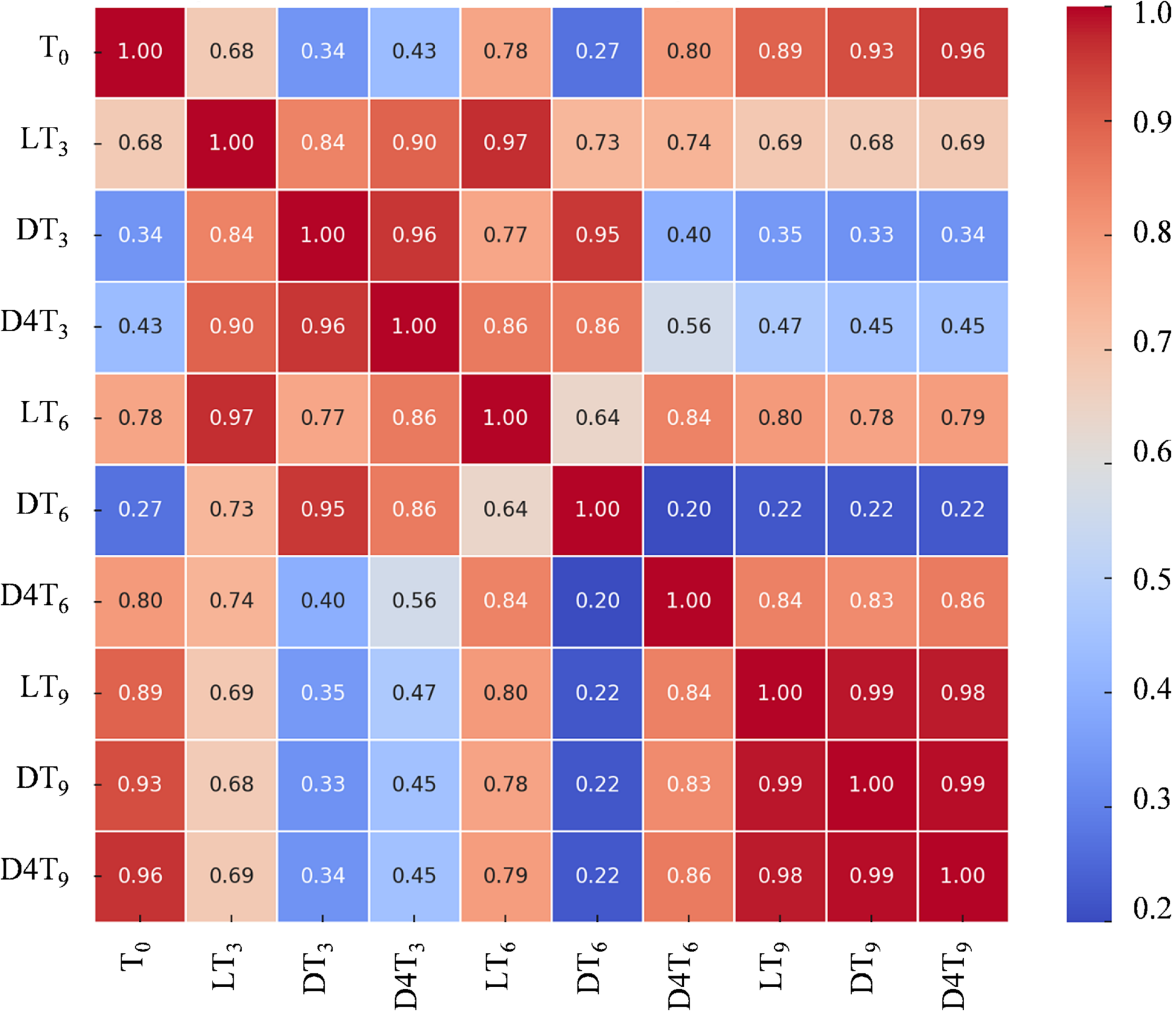
Heatmap of correlations between the different experimental conditions: light RT (L), dark RT (D) and dark 4°C (D4T) over time (T_0_-T_9_).

These results become even clearer when analyzed using a 2D correlation matrix of the phytochemical profiles under different experimental conditions (Figure 4).

The values of the first dimension are represented as the rows of the table, while those of the second dimension are displayed as columns. The color of each cell is proportional to the number of measurements that correspond to the dimensional values. This feature makes correlation heatmaps particularly effective for data analysis, as they facilitate the identification of patterns and highlight differences and variations within the dataset. Like a standard heatmap, a correlation heatmap is accompanied by a color bar, enhancing data readability and comprehension.

Based on these findings, it can be concluded that for short-term storage (up to 6 months), maintaining almonds at room temperature under normal light conditions is recommended to preserve their native polyphenolic profile. However, for storage periods exceeding 6 months, it is advisable to store them in the dark at a controlled temperature (4°C).

### 3.4 Evaluation of antioxidant activity during simulated storage conditions

To assess the free-radical scavenging activity of NS extracts at different time points (T_0_–T_9_) and under various storage conditions (light, dark, and dark at 4°C), both cell-free and cell-based models were employed.

The antioxidant properties were preliminarily investigated using four spectrophotometric and spectrofluorimetric assays, each based on different mechanisms and reaction environments: DPPH, TEAC, FRAP, and ORAC assays. Trolox was used as the reference standard, and the results were expressed as the concentration required to inhibit 50% of free radical activity (IC₅₀), with 95% confidence intervals provided in parentheses (Table 4).

Notably, the results, regardless of the assay used, perfectly align with the total phenolic content of the examined extracts (Table 3). T0, which exhibits the highest polyphenolic content, demonstrates significantly greater antioxidant properties (*p* < 0.001) than all other NS extracts, irrespective of time and storage conditions.

After 3 months, the LT_3_ sample displays significantly higher free-radical scavenging activity (*p* < 0.001) compared to DT_3_ and D4T_3_. Consistent with the observations on TPC (Table 3), a reversal of this trend is noted at T_6_, and even more so at T_9_. Although no statistically significant differences were found among the various storage methods, D4T_6_ exhibits the strongest antioxidant activity in terms of IC₅₀, followed by LT_6_ and DT_6_. At T_9_, according to the higher polyphenols content, D4T_9_ remains the sample with the highest antioxidant activity, followed by DT_9_ and LT_9_ (Table 5).

**Table 5:** The antioxidant activity of natural almond skin (NS) extracts was assessed at different time points (T0, T3, T6, and T9) and under various storage conditions, including light exposure (L), dark (D), and dark at 4°C (D4), using in vitro cell-free assays based on different mechanisms and reaction environments.

NS	DPPH	TEAC	FRAP	ORAC
	μg/ml			
T_0_	25.03 (21.31–29.40) ^a^	23.67 (19.42–26.46) ^b^	15.96 (12.64–20.14) ^c^	0.07 (0.06–0.09) ^a^
LT_3_	94.84 (77.17–116.56) ^d^	40.33 (33.41–48.69) ^d^	39.76 (32.83–48.15) ^d^	0.69 (0.56–0.86) ^e^
DT_3_	273.65 (244.56–298.32)	109.46 (89.68–133.61)	126.47 (100.63–158.96)	2.09 (1.62–2.71)
D4T_3_	227.38 (180.57–285.90)	74.57 (60.02–92.66)	109.30 (85.93–139.04)	1.20 (0.51–2.86)
LT_6_	58.37 (46.15–73.83)	23.63 (20.25–27.56)	27.48 (10.04–35.20)	0.17 (0.13–0.23)
DT_6_	66.01 (53.30–81.75)	26.22 (22.55–30.49)	31.81 (27.80–36.39)	0.29 (0.13–0.34)
D4T_6_	42.69 (38.54–56.78)	23.05 (17.14–25.58)	19.04 (15.26–26.26)	0.15 (0.10–0.22)
LT_9_	64.95 (50.05–84.30)	26.23 (18.51–34.24)	41.92 (33.38–52.65)	0.17 (0.14–0.20)
DT_9_	47.38 (40.44–50.55)	23.23 (21.31–25.49)	32.25(27.00–38.51)	0.13 (0.10–0.22)
D4T_9_	42.23 (34.26–52.05)	23.05 (21.18–25.22)	30.20 (22.62–42.24)	0.12 (0.10–0.25)
Trolox (μg/ml)	15.98 (12.74–20.05)	10.51 (8.30–13.29)	3.95 (1.67–9.32)	0.62 (0.13–1.87)

Results, expressed relative to the reference standard Trolox, represent the mean of three independent experiments performed in triplicate (*n* = 3). Data are reported as the concentration required to inhibit 50% of free radical activity (IC₅₀), with 95% confidence intervals provided in parentheses.

a *p < 0.001 vs. L, D and D4T3–9 NS extracts.*

b *p < 0.001 vs. L, D and D4T3 and D4T6 NS extracts.*

c *p ≤ 0.05 vs. LT3 and T9, DT3-9 and D4T3–9 NS extracts.*

d *p < 0.001 vs. D and D4T3.*

e *p < 0.001 vs. D4T3.*

## 4 Discussion

The present study systematically evaluated the impact of storage conditions on the microbiological safety of natural almonds as well as on the polyphenols content and antioxidant activity of almond skin extracts. It was revealed that environmental factors, including light exposure, dark, and refrigeration, while ensuring microbiological safety, significantly influence polyphenol degradation and transformation over time. These findings align with and expand upon previous literature research, offering a wider understanding of polyphenol stability in almonds and related almond-based products.

Rodriguez et al. (27) evaluated the influence of storage conditions on the microbial and mycotoxin stability of almonds, identifying significant differences after 3- and 9-months storage. In terms of aerobic mesophiles, six fungal genera—*Aspergillus, Cladosporium, Fusarium, Penicillium, Paecilomyces,* and *Talaromyces*—were detected, along with the presence of mycotoxins.

Gupta et al. (28) examined the impact of storage temperature on the microbiological quality of almond-supplemented *Paneer Kheer*, demonstrating that higher temperatures and prolonged storage negatively affected the product’s shelf life. Additionally, long-term storage of peeled almond kernels under high relative humidity has been shown to promote fungal growth, mycotoxin production, and rancidity (29). In our study, the humidity increases from week 24 onwards did not impact fungal growth. Various decontamination techniques, including ozone treatment, cold plasma, irradiation, and radiofrequency, have been explored to reduce microbial contamination in nuts and may be recommended to enhance overall shelf life (13).

Although *Salmonella* sp. was not detected in our study, several investigations have assessed its survival and thermal resistance in almonds after long-term storage (30–33), providing crucial insights for pathogen control processes.

Regarding polyphenols, the observed decrease in total phenolic compounds and flavonoids is consistent with existing literature, which identifies oxidation, hydrolysis, and enzymatic degradation as key factors affecting polyphenol retention (12, 16). Polyphenols are highly susceptible to degradation under room conditions, particularly when humidity and temperature are not tightly controlled. Interestingly, this study found that, after 3 months, light-exposed samples retained more polyphenols than those stored in complete dark, contradicting the assumption that light accelerates degradation. While polyphenols are generally considered light-sensitive, certain phenolic compounds may undergo stress-induced biosynthesis in response to moderate environmental stressors, including controlled light exposure (27). This could explain why LT_3_ samples retained higher polyphenol levels than DT_3_. However, prolonged exposure to uncontrolled light and oxygen may still lead to oxidative degradation, supporting the common recommendation to minimize direct light exposure in long-term storage (12).

At 6 months, a notable increase in polyphenol content was observed in refrigerated dark-stored samples. This finding is strongly supported by previous research, which observed that cold storage (1°C to 10°C) activates the phenylpropanoid pathway, leading to an increase in phenolic biosynthesis (17). The enzymatic activity of phenylalanine ammonia-lyase (PAL), a key regulator of polyphenol biosynthesis, has been found to increase under cold stress, explaining why refrigeration may aid in polyphenol preservation and even stimulate their synthesis. At 9 months, the highest total phenolic compounds and flavonoids levels were recorded in refrigerated dark storage, reinforcing findings from previous studies that cold, dark conditions offer optimal long-term preservation of polyphenols (27). However, the unexpected higher total phenolic compounds in DT_9_ compared to LT_9_ suggests that selective degradation of flavonoids and potential conversion into alternative bioactive derivatives may have occurred. The literature notes that oxidation and polymerization reactions can alter phenolic structure over time, sometimes leading to the formation of more stable or bioactive derivatives (16, 34). Furthermore, the increase in relative humidity recorded in the present study from week 24 onwards may have also influenced enzymatic activity, promoting these processes.

The study identified 27 distinct polyphenolic compounds, including 4 phenolic acids and 23 flavonoids, each exhibiting distinct stability patterns. These findings agree with previous research highlighting compound-specific responses to storage conditions (17, 34). Chlorogenic acid exhibited a significant decline at T_3_, with a recovery at T_6_ in refrigerated samples (D4T_6_). This aligns with prior studies indicating that chlorogenic acid is highly susceptible to oxidation but may become more extractable under cold storage conditions (27). Quercetin derivatives followed a similar trend, decreasing initially and partially recovering in refrigerated samples at T_9_ (D4T_9_), suggesting potential enzymatic conversion into bioavailable metabolites under cold stress. Epicatechin displayed significant depletion at T_3_ but demonstrated partial restoration over time, mirroring findings that oxidation reactions during storage degrade catechins but can also lead to polymerized procyanidin forms with altered bioactivity (16). Isorhamnetin-3-O-glucoside, one of the most abundant flavonoids, exhibited fluctuating levels across storage conditions. This observation is supported by research indicating that isorhamnetin glycosides undergo structural modifications depending on environmental stress factors, including enzymatic activity and microbial interactions (27).

The antioxidant activity, as evaluated by DPPH, TEAC, FRAP, and ORAC assays, exhibited a strong correlation with total phenolic compounds levels, supporting the well-established relationship between polyphenolic concentration and free radical-scavenging ability. The highest antioxidant activity was observed at T_0_, followed by a significant decline at T_3_. However, LT_3_ retained higher antioxidant potential than DT_3_, again suggesting that moderate light exposure may play a role in stabilizing or activating certain phenolic compounds. By T_6_, a partial recovery in antioxidant activity was noted, with D4T_6_ samples exhibiting the highest radical-scavenging capacity. This correlates with previous research findings, which reported that cold storage can enhance the antioxidant properties of polyphenols through enzymatic stress responses (17). At T_9_, D4T_9_ retained the highest antioxidant activity, further confirming that refrigerated dark storage is the most effective condition for long-term bioactivity preservation.

The findings of this study provide valuable insights into the optimal storage strategies for almond-based products, complementing and expanding upon existing literature. Short-term products such as almond-infused beverages and energy bars benefit from room-temperature storage with controlled light exposure, while long-term products like almond-based supplements should be stored in refrigerated dark conditions to retain bioactivity. Polyphenol-rich extracts used in anti-aging formulations should prioritize cold storage to maintain antioxidant stability, with encapsulation technologies such as liposomes further protecting sensitive compounds from oxidation. For long-term stability, almond polyphenol extracts should be stored under refrigerated, dark conditions, and liquid formulations should incorporate stabilizing antioxidants such as vitamin C or vitamin E to mitigate oxidative degradation. Fresh extracts are best used within the first 3 months for maximum bioactive content, while almond oils and baking additives should be stored at 4°C to retain their functional properties.

This study substantiates and expands upon existing literature, confirming that storage conditions exert a profound influence on polyphenol retention and antioxidant activity in almond skin extracts. The correlation between total phenolic compounds and flavonoids, and antioxidant potential aligns well with previous research, emphasizing that cold, dark storage is the optimal long-term preservation method. Additionally, the findings provide wider insights into compound-specific degradation patterns and enzymatic metabolic transformations, supporting the need for tailored storage solutions across various industries. These results reinforce the notion that strategic storage conditions are critical for maintaining the nutraceutical and functional properties of almond-derived products, providing actionable guidance for both industrial applications and consumer storage recommendations. These findings agree with previous research emphasizing the necessity of controlled humidity, temperature, and packaging choices in optimizing the bioavailability and functionality of polyphenols in tree nuts and their by-products (12, 16, 27, 34).

## 5 Conclusion

This study examines the complex dynamics of polyphenol degradation in almond skin extracts and highlights the importance of tailored storage strategies, also in relation to microbiological safety. While polyphenol content and antioxidant activity inevitably diminish over time, strategic preservation methods can significantly attenuate these losses. For short-term storage (≤ 6 months), room temperature with light exposure is sufficient, whereas long-term storage (> 6 months) necessitates refrigerated, dark conditions to optimize phenolic content and antioxidant retention. Different product formulations demand specific storage considerations, and manufacturers should adapt storage protocols accordingly to maximize product efficacy and shelf life. The findings presented herein provide a foundational framework for industries spanning food, cosmetics, and pharmaceuticals, facilitating evidence-based decision-making in almond-based product development and preservation strategies. Further research may focus on exploring the molecular mechanisms underpinning polyphenol transformation under varying storage conditions, to refine and enhance preservation methodologies.

## Data Availability

The raw data supporting the conclusions of this article will be made available by the authors, without undue reservation.
